# Low Birth Weight and Risk of Progression to End Stage Renal Disease in IgA Nephropathy—A Retrospective Registry-Based Cohort Study

**DOI:** 10.1371/journal.pone.0153819

**Published:** 2016-04-19

**Authors:** Paschal Ruggajo, Einar Svarstad, Sabine Leh, Hans-Peter Marti, Anna Varberg Reisæther, Bjørn Egil Vikse

**Affiliations:** 1 Department of Internal Medicine, MUHAS, Dar es Salaam, Tanzania; 2 Department of Clinical Medicine, University of Bergen, Bergen, Norway; 3 Department of Medicine, Haukeland University Hospital, Bergen, Norway; 4 Department of Pathology, Haukeland University Hospital, Bergen, Norway; 5 Medical Birth Registry of Norway, Norwegian Institute of Public Health, Bergen, Norway; 6 Department of Transplantation Medicine, Rikshospitalet, Oslo University Hospital, Oslo, Norway; 7 Department of Medicine, Haugesund Hospital, Haugesund, Norway; Osaka University, Graduate School of Medicine, JAPAN

## Abstract

**Background:**

Low Birth Weight (LBW) is a surrogate for fetal undernutrition and is associated with impaired nephron development in utero. In this study, we investigate whether having been born LBW and/or small for gestational age (SGA) predict progression to ESRD in IgA nephropathy (IgAN) patients.

**Study Design:**

Retrospective registry-based cohort study.

**Settings & Participants:**

The Medical Birth Registry has recorded all births since 1967 and the Norwegian Renal Registry has recorded all patients with ESRD since 1980. Based on data from the Norwegian Kidney Biopsy Registry we included all patients diagnosed with IgAN in Norway from 1988–2013. These registries were linked and we analysed risk of progression to ESRD associated with LBW (defined as birth weight less than the 10^th^ percentile) and/or SGA (defined as birth weight less than the 10^th^ percentile for gestational week) by Cox regression statistics.

**Results:**

We included 471 patients, of whom 74 developed ESRD. As compared to patients without LBW, patients with LBW had a hazard ratio (HR) of 2.0 (95% confidence interval 1.1–3.7) for the total cohort, 2.2 (1.1–4.4) for males and 1.3 (0.30–5.8) for females. Corresponding HRs for SGA were 2.2 (1.1–4.2), 2.7 (1.4–5.5) and 0.8 (0.10–5.9). Further analyses showed that as compared to patients with neither LBW nor SGA, patients with either SGA or LBW did not have significantly increased risks (HRs of 1.3–1.4) but patients who were both LBW and SGA had an increased risk (HR 3.2 (1.5–6.8).

**Limitation:**

Mean duration of follow-up only 10 years and maximum age only 46 years.

**Conclusion:**

Among IgAN patients, LBW and/or SGA was associated with increased risk for progression to ESRD, the association was stronger in males.

## Introduction

Brenner hypothesized in 1988 that adverse intrauterine environment, for example due to placental insufficiency or maternal malnutrition, was associated with impaired nephron development and increased risk of hypertension and progressive kidney disease in adult life[1]. Low birth weight (LBW) is the most accessible marker of adverse intrauterine environment[1] and studies have shown strong associations with fewer and larger glomeruli[2,3], increased risk of hypertension[4,5], albuminuria [6,7] and progressive chronic kidney disease[8,9]. It is possible that SGA, defined as low birth weight for gestational age, is a better marker for adverse intrauterine environment and studies have shown associations with lower GFR [10,11]. Previous studies have suggested LBW, SGA and preterm birth to be associated with reduced estimated and measured GFR [12–14].

IgA nephropathy (IgAN) is the most frequently occurring primary idiopathic glomerulonephritis worldwide [15–17]. The clinical phenotype of IgAN range from stable and asymptomatic to chronic and progressive renal failure [18–20] and several studies have investigated clinical and histopathological variables as risk factors for progressive disease [21–25]. Due to its chronic course it is possible to look upon IgAN as a model disease for chronic kidney disease in general and the advantages of this might be that IgAN patients generally are younger, have fewer confounding comorbidities while on the other hand having a high rate of progressive disease. Specifically, for the investigation of the Brenner hypothesis, better access to birth related data in these younger patients is also of benefit. As described above, LBW might be a risk factor for progressive IgAN as LBW is associated with fewer and larger glomeruli [2,3]. Indeed a previous study by Tsuboi et al demonstrated that lower glomerular density predicted the long-term prognosis of IgAN[26]. Also in support of this, low birth weight was associated with higher rates of progressive disease in a small study of children with IgAN[27]. More and larger studies are however needed to explore this further.

In the present study we used data from Norwegian Registries and analyzed whether adverse birth-weight related variables are associated with development of end-stage renal disease (ESRD) in patients diagnosed with IgAN in Norway in the period 1988–2013. We first analyzed whether LBW predicted progression to ESRD, but also analyzed the effects of SGA and preterm birth, and combinations of these factors. Our main hypothesis was that LBW and SGA predict progression to ESRD, in line with the Brenner hypothesis.

## Material and Methods

Since 1967, the Medical Birth Registry of Norway has registered extensive medical data on all births in Norway (total population of 5.1 million) [28]. The form is completed by the attending midwife and doctor. Since 1980, the Norwegian Renal Registry has registered data on all patients in Norway who develop ESRD (defined as starting chronic dialysis treatment or undergoing renal transplantation). The Norwegian Kidney Biopsy Registry has registered clinical and morphologic data for all patients who have had a kidney biopsy performed in Norway since 1988. All kidney biopsies in this study were evaluated by an experienced nephropathologist. The data from all registries were available until December 2013 and data were linked using the 11-digit unique national identification number.

We included all patients diagnosed with IgA nephropathy in the Norwegian Kidney Biopsy Registry between 1988 and 2013 and who were born in Norway after 1967 and had their birth data registered in the Medical Birth Registry. The study was approved by the Regional Ethics Committee of Norway. Patient records/information was anonymized and de-identified prior to analysis. This investigation abides to Declaration of Helsinki.

### Exposure Variables

LBW was defined as birth weight less than the 10^th^ percentile for gender (2930g for male; 2690g for female). Based on total data from the Medical Birth Registry, different cut-offs were tested in separate analyses (For male and female gender respectively the 25^th^ percentile was defined as 3233g and 3050g, the 20^th^ percentile as 3168g and 2960g, the 15^th^ percentile as 3070g and 2910g and the 7.5^th^ percentile as 2860g and 2640g). From 1967 through 1998, gestational age was based on the last menstrual period and from 1999 onward on routine ultrasonographic examination in gestational weeks 17 through 20. Preterm birth was defined as a gestational age less than 37 weeks. Based on national data on birth weight, gestational week, gender and plurality, a z-score denoting standard deviation from mean of birth weight for each week of gestational age was calculated for each patient by the Medical Birth Registry [29,30]. Small for gestational age (SGA) was defined as birth weight less than the 10^th^ percentile for gestational week in the study population (defined by z-score less than -1.2900 for male and -1.5280 for female gender). In separate analyses, different cut-offs for birth weight for gestational age (defined by z-score), separately for gender, were tested. The respective z-scores for males and females for the 25^th^ percentile were defined as -0.7400 and -0.9600, for 20^th^ percentile as -0.8680 and -1.1620, for 15^th^ percentile as -1.1120 and -1.3360 and for 7.5^th^ as -1.5000 and -1.6620. Maternal preeclampsia was defined as increased BP and proteinuria after 20 weeks of gestation (BP ≥140/90)[31].

Recorded standard clinical and laboratory tests were performed at the time of kidney biopsy. Estimated glomerular filtration rate (eGFR) was calculated using the IDMS-traceable CKD-EPI equation [32] using the serum creatinine values (unit micromoles/L, converted to mg/L for eGFR calculation) recorded at the time of biopsy (All patients were assumed to be of white race). The CKD-EPI is calculated as; eGFR = 141 x Min (Serum Creat/k,1)^α^ x Max(Serum Creat/k,1)^-1.209^ x 0.993^Age^ x 1.018 (if female); where k = 0.7 for females and 0.9 for males, α = 0.329 for females and 0.411 for males [33]. For patients who had a kidney biopsy performed before year 2005, their serum creatinine levels were reduced by 5% to standardize them to IDMS-traceable levels [33]. For use in the present study we defined three categories of eGFR as: >60, 30–60 and <30 ml/min/1.73m^2^. Proteinuria had been registered as grams per 24 h, grams per litre and milligrams per millimole creatinine and as stix results. Estimates for proteinuria per 24h were calculated as described previously [34], three categories of <1, 1–3 and > = 3 grams/24h were used in the analyses. Systolic blood pressure was reported in three categories based on blood pressure level: <140, 140–159 or > = 160 mmHg, cut-offs for diastolic BP were 90 and 100 mmHg. The following histopathological parameters were used; proportion of sclerosed glomeruli, proportion of glomeruli with crescents (cellular or fibrocellular), grade of interstitial fibrosis (categorized as mild, moderate or severe if the fibrosis involved <25%, 25–50% or more than 50% of the cortical area respectively) and grade of tubular atrophy which was (categorized as mild, moderate or severe if the tubular atrophy involved <25%, 25–50% or more than 50% of the cortical area). Treatment data (type of medications, dose and duration) were not available, see discussion for a description of standard treatment in Norway.

### Outcome Variables

The outcome was development of ESRD defined as the date of starting chronic dialysis treatment or undergoing renal transplantation. Individuals who did not develop ESRD were followed until December 31, 2013.

### Statistical Analyses

Data were analysed in a cohort design with birth-weight related variables as exposure and ESRD as outcome variables. Hazard ratio estimates associated with selected risk factors for ESRD were obtained by Cox regression analyses. Assumptions of proportional hazard were tested by log-minus-log plots and the assumptions were met. Analyses were performed for the total cohort, but also separately for male and female. Associations between birth weight related variables and clinical and histopathological variables at time of biopsy were investigated but in the present paper we did not focus on the effects of the latter variables on risk of ESRD as these have been thoroughly described previously[35]. Due to low number of endpoints, we chose to only perform adjusted analyses for eGFR. The analyses were performed with the statistical package SPSS 21 (SPSS, Chicago, IL). Unless otherwise noted, values are reported as means (standard deviation) or hazard ratio estimates (95% confidence intervals). P-values < 0.05 were considered statistically significant, and all tests were two tailed.

## Results

A total of 471 patients (70.8% males) were included in our study, of whom 74 (15.7%) developed ESRD. Mean age at biopsy was 23.8 (7.7) years, mean duration of follow-up after biopsy was 10.3 (6.7) (range 0.08–25.8) years, mean age at ESRD was 29.3 (7.0) years and mean age at end of follow-up for those who did not develop ESRD was 35.1 (8.1) years. Of the included patients, 10.2% were categorized as having had LBW and 9.6% as SGA.

At the time of kidney biopsy, IgAN patients born with LBW or SGA had comparable clinical and pathological characteristics as those born without LBW (Table 1). When the same analysis was repeated gender-wise, males had significantly higher systolic blood pressure (132 mmHg vs 123 mmHg, p-value < 0.001) and diastolic blood pressure (80 mmHg vs 76 mmHg, p-value 0.01) than females. There was no gender difference observed in the other clinicopathological characteristics, including estimated GFR. As expected, patients born with LBW or SGA more often were born preterm, patients born SGA also had a statistically significant higher likelihood of being born in a preeclamptic pregnancy.

**Table 1:** Cohort characteristics at the time of IgAN diagnosis stratified by Low Birth Weight and Small for Gestational Age, Norway 1967–2013.

	LBW		SGA	
Clinicopathological characteristics	No	Yes	No	Yes
N (%)	423	48 (10.2)	405	45(9.6)
N (%) male	298 (70.4)	34 (70.8)	283 (69.9)	32 (71.1)
Age (years)	23.8±7.6	24.7±8.6	23.8± 7.7	25.6±8.3
Systolic BP (mmHg)	129.1±21.0	128.8±20.7	128.7±21.4	130.3±18.2
Diastolic BP (mmHg)	78.5±14.1	77.6±14.6	78.5 ± 14.4	76.6 ±13.0
eGFR (ml/min/1.73m^2^)	100.2±50.1	92.4±38.5	100.7±50.8	87.7±38.3
Urinary protein (g/d)	2.0±2.4	2.6±2.6	2.0±2.5	2.6±2.8
Proportion sclerosed glomeruli (%)	0.1±0.2	0.1±0.2	0.1±0.2	0.1±0.2
Proportion with glomerular crescents (%)	1.5±6.0	1.1±3.1	1.4±5.8	1.9±5.6
Grade of interstitial fibrosis ^b^	0.8±0.7	1.0±0.7	0.8±0.7	0.8±0.8
Grade of tubular atrophy ^c^	0.7±0.8	0.9±1.0	0.7±0.8	0.8±0.9
Pre-eclampsia in the mother (%)	7 (1.7)	3 (6.3)	3 (0.7)	6 (13.3) ^**a**^
N (%) birth weight <10^th^ percentile (LBW)	0%	100% (by def.)	18(4.4)	24 (53.3) ^**a**^
N (%) gestational age <37 weeks	8 (2.0)	19 (41.3)	24 (5.9)	3 (6.7)
N (%) birth weight <10^th^ percentile for gestational age (SGA)	19 (4.7)	26 (56.5)	0%	100% (by def.)

^a^ p<0.001

^b^ Grade of interstitial fibrosis was categorized as mild, moderate or severe if the fibrosis involved <25%, 25–50% or more than 50% of the cortical area respectively.

^c^ Grade of tubular atrophy was categorized as mild, moderate or severe if the tubular atrophy involved <25%, 25–50% or more than 50% of the cortical area respectively.

In separate analyses of male and female patients, SGA was significantly associated with higher urinary protein excretion (3.0 vs 1.9 gram/24h; p = 0.04) in males but not females. Analyses for other clinical and histopathological variables at the time of biopsy showed no significant associations. Comparing clinicopathological characteristics in Table 1 between those with both LBW and SGA to those with neither of them showed no significant differences.

As expected from previous studies [35], patients who developed ESRD had lower eGFR, higher blood pressure, higher urinary protein excretion and higher grade of interstitial fibrosis or tubular atrophy and had a higher risk of developing ESRD. The present study does not focus on these data, but for completeness, details are given in S1 Table and S2 Table.

### Birth weight related variables and the risk of developing ESRD

Compared to patients with birth weight above the 10^th^ percentile, LBW was significantly associated with higher risk of developing ESRD, HR 2.0 (95% CI 1.1–3.7; p = 0.03) (Table 2). Similarly, SGA was also significantly associated with ESRD; HR 2.2 (1.2–4.2); p = 0.02).

**Table 2:** Risk of ESRD according to whether the IgAN patients had LBW, SGA or preterm birth, separate analyses for male and female, Norway 1967–2013.

			Unadjusted model		Adjusted model^a^	
	N total	N ESRD	HR (95% CI)	p-value	HR (95% CI)	p-value
**Total**	471	74				
Not LBW	423	62	1.0 (ref)		1.0 (ref)	
LBW	48	12	2.0 (1.1–3.7)	0.03	1.4 (0.69–2.7)	0.4
Not SGA	405	57	1.0 (ref)		1.0 (ref)	
SGA	45	11	2.2 (1.1–4.2)	0.02	1.4 (0.67–3.0)	0.4
Not Preterm	423	62	1.0 (ref)		1.0 (ref)	
Preterm	27	6	1.5 (0.65–3.5)	0.3	0.95 (0.38–2.4)	0.9
**Male**						
Not LBW	298	48	1.0 (ref)		1.0 (ref)	
LBW	34	10	2.2 (1.1–4.4)	0.02	1.1 (0.52–2.4)	0.8
Not SGA	283	43	1.0 (ref)		1.0 (ref)	
SGA	32	10	2.7 (1.4–5.5)	0.005	1.4 (0.64–3.2)	0.4
Not Preterm	295	48	1.0 (ref)		1.0 (ref)	
Preterm	20	5	1.6 (0.64–4.0)	0.3	0.74 (0.26–2.1)	0.7
**Female**						
Not LBW	125	14	1.0 (ref)		1.0 (ref)	
LBW	14	2	1.3 (0.30–5.8)	0.7	1.4 (0.32–6.4)	0.6
Not SGA	122	14	1.0 (ref)		1.0 (ref)	
SGA	13	1	0.8 (0.10–5.9)	0.8	0.8 (0.11–6.3)	0.9
Not Preterm	128	14	1.0 (ref)		1.0 (ref)	
Preterm	7	1	1.1 (0.14–8.4)	0.9	1.0 (0.14–8.0)	1.0

^a^Adjusted for estimated Glomerular Filtration in 3 categories (i.e. > 60, 30–59 and < 30 ml/min respectively).

**Fig 1:**
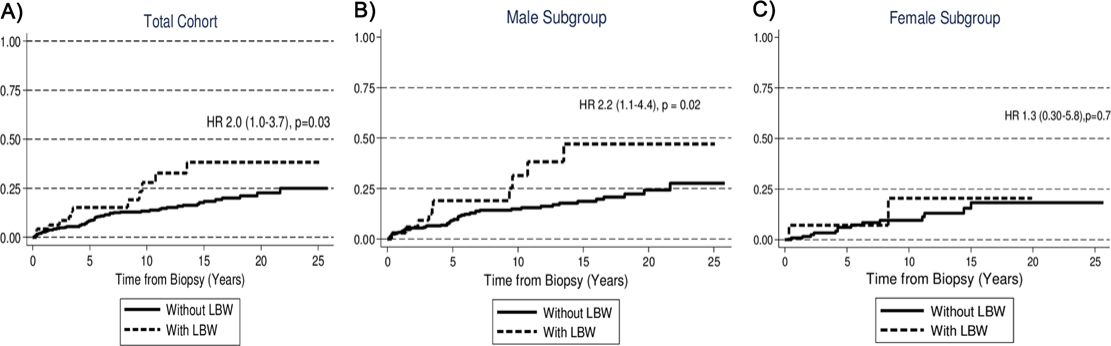
Cumulative Probability of ESRD in patients with IgAN according to whether or not the patient had LBW.

**Fig 2:**
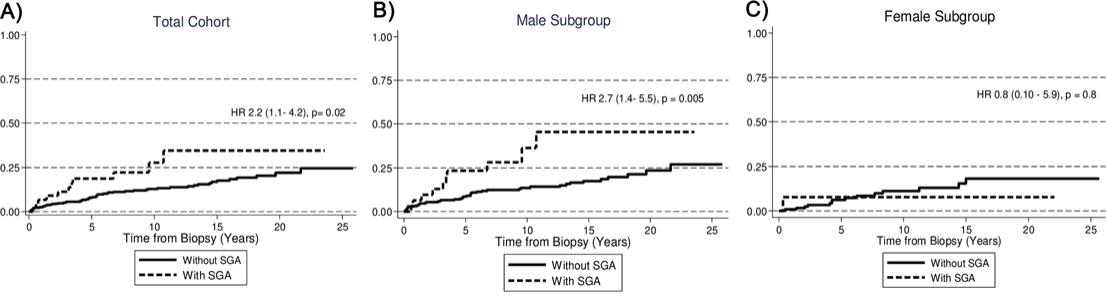
Cumulative Probability of ESRD in patients with IgAN according to whether or not the patient had SGA.

When the analyses were done separately for male and female patients, both SGA and LBW remained statistically significant predictors of ESRD in males, but there was no excess risk in females (Table 2 and Figs 1 and 2). After adjustments for eGFR, neither LBW nor SGA were significantly associated with risk of ESRD. A similar analysis for preterm birth, showed a non-significant higher risk of ESRD for the total cohort and for both gender subgroups.

To explore these associations further, we analyzed risk of developing ESRD by decremental birth weight and birth weight according to the gestational age (Table 3). In these analyses we categorized birth weight and birth weight for gestational age below 25^th^, 20^th^, 15^th^, 10^th^ and 7.5^th^ percentile cut-offs. For birth weight, the hazard ratio was statistically significant for the 15^th^ and 10^th^ percentile but not significant for the 7.5^th^ percentile. For birth weight for gestational age, the hazard ratio increased with lower cut-off and was statistically significant for the 10^th^ and 7.5^th^ percentile cut-off, the hazard ratio was highest when using the 7.5^th^ percentile. A separate analysis using a cut-off of 2.5 kg (conforming to the WHO global LBW threshold) showed hazard ratio of 1.0 (0.31–3.1) for the total cohort, 1.24 (0.30–5.1) for males and 0.81 (0.11–6.1) for females; however, analysis using this cut-off value was limited by the low number of patients (only 20 patients had birth weight less than 2.5 kg, of whom 3 developed ESRD).

**Table 3:** Risk of ESRD among IgAN patients analysed at different percentile cut-offs for birth weight and birth weight for gestational age.

			Unadjusted		Adjusted ^a^	
	N total	N ESRD	HR (CI)	p-value	HR (CI)	p-value
**Birth weight percentile**^**b**^						
≥25^th^ perc	352	52	1.0(ref)	1.0 (ref)	
<25^th^ perc	119	22	1.3(0.76–2.1)	0.4	0.96(0.57–1.6)	0.9
≥20^th^ perc	377	53	1.0 (ref)		1.0 (ref)	
<20^th^ perc	94	21	1.7(1.0–2.8)	0.05	1.2(0.69–2.0)	0.5
≥15^th^ perc	401	58	1.0 (ref)		1.0 (ref)	
<15^th^ perc	70	16	1.8(1.0–3.2)	0.04	1.1 (0.58–2.0)	0.8
≥10^th^ perc^c^	423	62	1.0 (ref)		1.0 (ref)	
<10^th^ perc^c^	48	12	2.0(1.1–3.7)	0.03	1.4(0.69–2.7)	0.4
≥7.5^th^ perc	437	66	1.0 (ref)		1.0 (ref)	
<7.5^th^ perc	34	8	1.6(0.78–3.4)	0.2	1.1(0.48–2.6)	0.8
**Birth weight for gestational age percentile**^**d**^						
≥ 25^th^ perc	336	49	1.0 (ref)		1.0(ref)	
< 25^th^ perc	114	19	1.2(0.68–2.0)	0.6	1.0(0.57–1.8)	1.0
≥ 20^th^ perc	360	52	1.0(ref)		1.0(ref)	
< 20^th^ perc	90	16	1.3 (0.76–2.3)	0.3	1.2(0.66–2.2)	0.5
≥ 15^th^ perc	383	56	1.0 (ref)		1.0(ref)	
<15^th^ perc	67	12	1.3 (0.69–2.4)	0.4	1.0(0.5–2.1)	1.0
≥10^th^ perc^c^	405	57	1.0 (ref)		1.0(ref)	
<10^th^ perc^c^	45	11	2.2 (1.1–4.2)	0.02	1.4(0.67–3.0)	0.4
≥7.5^th^ perc	416	59	1.0 (ref)		1.0 (ref)	
<7.5^th^ perc	34	9	2.5(1.2–5.1)	0.01	1.4(0.62–3.4)	0.4

^a^Adjusted for estimated Glomerular Filtration in 3 categories (i.e. > 60, 30–59 and < 30 ml/min respectively)

^b^ for males, birth weight percentile < 25th, <20th, <15th, <10th and <7.5th percentile was defined as < 3.2325 kg, 3.1680 kg, 3.0695 kg, 2.9300 kg and 2.8597 kg respectively; for females, corresponding birth weights were 3.0500 kg, 2.9600 kg, 2.9100 kg, 2.6900 kg and 2.6400 kg respectively.

^c^ Identical analysis as in Table 2, included for completeness.

^d^ for males, birth weight for gestational age z–scores corresponding to < 25th, <20th, <15th, <10th and <7.5th were -0.7400,-0.8680,-1.1120,-1.2900 and -1.5000 respectively. For females, corresponding z-scores were -0.9600,-1.1620, -1.3360, -1.5280 and -1.6620.

### Single versus multiple adverse birth-weight related outcomes and risk of ESRD

From the analyses presented in Tables 2 and 3, SGA was associated with slightly higher hazard ratios for ESRD as compared to LBW. Preterm birth was on the other hand not associated with development of ESRD. To explore this further, we analyzed the combined effect of SGA and/or LBW on the risk of developing ESRD (Table 4). In these analyses, patients who were either SGA or LBW had no increased risk, but patients who were both SGA and LBW had a significantly higher HR of 3.2 (1.5–6.8), p = 0.002 for the total cohort and a HR of 3.6 (1.6–8.2), p = 0.02 for males (Table 4), the number of patients in subgroups were however low and confidence intervals were wide.

**Table 4:** Risk of ESRD among IgAN patients stratified by LBW, SGA or the combination of these, (gender-wise).

			Unadjusted model		Adjusted model^a^	
	N total	N ESRD	HR (95% CI)	p-value	HR (95% CI)^a^	p-value
**Total**						
Not SGA or LBW	385	53	1.0 (ref)		1.0 (ref)	
SGA, not LBW	19	3	1.3 (0.39–4.0)	0.7	0.74(0.18–3.0)	0.7
Not SGA, LBW	20	4	1.4 (0.51–3.9)	0.5	1.0 (0.36–2.8)	1.0
SGA and LBW	26	8	3.2 (1.5–6.8)	0.002	2.0 (0.87–4.8)	0.1
**Male**						
Not SGA or LBW	270	40	1.0 (ref)		1.0 (ref)	
SGA, not LBW	13	3	1.9 (0.57–6.0)	0.3	0.89 (0.21–3.7)	0.9
Not SGA, LBW	13	3	1.6 (0.50–5.3)	0.4	0.74 (0.22–2.5)	0.6
SGA and LBW	19	7	3.6 (1.6–8.2)	0.002	1.8 (0.70–4.6)	0.2
**Female**						
Not SGA or LBW	115	13	1.0 (ref)		1.0 (ref)	
SGA, not LBW	6	0	0.0	1.0	0.0	1.0
Not SGA, LBW	7	1	1.0 (0.13–7.8)	1.0	1.0 (0.14–8.1)	1.0
SGA and LBW	7	1	1.8 (0.23–13.6)	0.6	2.2(0.28–17.1)	0.5

^a^ Adjusted for estimated Glomerular Filtration in 3 categories (i.e. > 60, 30–59 and < 30 ml/min respectively)

Repeated analyses testing the combined effect of preterm birth and/or LBW showed that LBW in preterm birth was not associated with higher hazard ratio of ESRD than LBW in term birth [2.2(0.97–5.2) vs 2.1 (0.89–4.5)]. Identical analysis for the combined effect of preterm birth and/or SGA did on the other hand show a stronger effect of SGA in preterm as compared to term births (10.8 (2.6–45) vs 1.9 (0.93–3.1) (results not shown).

## Discussion

In this comprehensive, registry-based study, we have shown that LBW was significantly associated with increased risk of progression to ESRD in IgAN. Having been born SGA, indicating intrauterine growth restriction, was also associated with ESRD. These effects were significant in the total cohort and in males, but we could not find evidence of increased risk in females. Further analyses showed that patients with combined LBW and SGA have significantly increased risk of developing ESRD as compared to patients with only one of the markers. After adjustments for eGFR at time of diagnosis, the birth weight related variables were no longer associated with progression to ESRD.

As described in the introduction, previous studies have shown associations between birth-weight related variables and increased risk for all-cause ESRD in the total population [8,36,37]. In a previous Norwegian study, LBW was also associated with higher risk of ESRD due to glomerular disease, of which IgAN was an important group [8]. The present study has added the information that LBW is a risk marker for progressive IgAN. This has to our knowledge never previously been shown in IgAN, although a previous study showed that children with IgAN who were born with LBW had significantly higher proportion of sclerotic glomeruli than children with normal birth weight [27]. Another study indicated higher rates of relapsing minimal change disease in children born with LBW[38]. LBW is the most used marker of intrauterine growth retardation and has been shown to be associated with a reduced number of enlarged glomeruli [2,3,39], salt sensitivity of blood pressure [40,41], higher blood pressure [42,43], microalbuminuria and low glomerular filtration rate [11], all markers that could be associated with higher risk of progressive renal failure [44,45]. Taken together, this suggests that LBW may be strongly associated with increased rates of progressive renal disease.

During nephrogenesis, week 9 to 36 in utero, the number of formed nephrons correlates with increase in fetal weight. LBW is the most accessible marker of adverse intrauterine environment [1], birth weight may however be affected both by duration of gestation and rate of fetal growth [46]. A previous histomorphometric study deduces that about 260,000 nephrons are formed for every 1kg increase in fetal weight in utero [3]. It can also be calculated that about twenty thousand nephrons are formed per week of gestation, and of these about 60% are formed during the third trimester [47]. Prematurity leads to 'oligonephropathy' with quantitative and qualitative alteration of nephron formation [48] and also LBW and SGA are associated with reduced nephron numbers in humans [1]. SGA may be a better marker of intrauterine growth restriction as LBW at short gestational age may be physiologically normal. In a previous Norwegian study that investigated risk of all cause ESRD, LBW was associated with slightly higher hazard ratio of ESRD than SGA[8].

In this current study, SGA was on the other hand associated with slightly higher hazard ratio than LBW. The finding that SGA might be a strong risk marker have been indicated by previous studies demonstrating increased risk for development of renal disease and hypertension [49,50], lower renal drug clearance [12], having smaller kidneys at birth and impaired kidney growth in early childhood [51]. An important observation was however that patients born with both LBW and SGA had much higher risk for developing ESRD than those born with only SGA or LBW, patients with only one marker did not have a statistically significant increased risk. In this study, we did not observe any significant difference in clinicopathological characteristics when we compared those who had combined LBW and SGA to those who had neither of them. Zidar et al on the other hand found higher mean percentage of sclerotic glomeruli among those with previous IUGR [52]. Our study indicate that future studies of intrauterine growth restriction and adult renal disease need to investigate both the birth weight as well as the birth weight in relation to gestational age. Patients with both LBW and SGA and renal disease may also need closer follow-up.

In the paragraph above we discussed how a lower number of nephrons could represent a lower reserve capacity and predispose to progressive renal disease. It is however also possible that intrauterine nutritional imbalances could program immunological function or risk that may persist throughout life [53]. Previous studies have suggested that early life perturbations of the immune system may be associated with lifelong increased risk for autoimmune and allergic diseases as well as chronic inflammatory conditions such as diabetes, cardiovascular diseases, metabolic syndrome and cancer [54–56]. Furthermore, prolonged, impairment of cell-mediated immunity is more common among LBW infants [57]. In relation to kidney diseases, Hoy et al found that low birth weight in the aborigines in Australia predisposed to post-infectious glomerulonephritis [58], this could however also have been explained by higher rates of poverty which highly correlates with both malnutrition and increased susceptibility to infectious diseases. Using the same definition of LBW as in a previous population-based study by us, we found that 11.9% of our IgAN patients would be categorised as LBW [59]. This is slightly higher than the 9.9% in the previous study[59], but this was not statistically significant. Thus, IgAN patients seem to have the same prevalence of LBW as the population and this argues against LBW being a significant risk marker for development of IgAN through immunological mechanisms.

Adjusted analysis showed that the association between LBW/SGA and progression to ESRD was lost after adjustments for eGFR. The most likely explanation for this is that eGFR is a much stronger risk factor for ESRD than LBW or SGA. It might however also imply that reduced GFR itself may be a consequence of being born SGA or LBW [12]. In the present study, males born with SGA had lower eGFR at time of diagnosis than males without SGA. It is still unclear whether LBW or SGA should be taken into consideration when treating patients with IgAN or planning studies of IgAN, but we would argue that more knowledge is needed and that there could be important effects that could be uncovered in larger or more targeted studies.

We observed that the association between LBW and increased risk of ESRD was significant and strong in males but not in females. Globally, gender distribution in IgA nephropathy varies widely in different populations with more preponderance towards male gender ranging from a male: female ratio of less than 2:1 in Japan [60] to 6:1 in the in the Western countries being highest in the in Northern Europe [61–63]. Recent registry-based data on IgAN in Japan[64] and Korea[65] have shown no appreciable gender difference as contrasted to data from the Norwegian Kidney Biopsy Register in which male comprised of 74% of all IgAN cases[25]. This wide range however may in part reflect the observed difference in subjective clinical indications and thresholds of performing a kidney biopsy among nephrologists in different countries, or other genetic/environmental differences between different areas of the world that have no clinical explanation so far[66]. The statistical analysis in our analyses may however be polarized towards showing stronger effect in males due to compromised statistical power in females. Previous studies however, have suggested that females may be protected against the detrimental effects of LBW on progressive renal disease [67–69], although renal clearance studies could not show a clear effect modification on the associations between LBW and reduced renal function [12,70,71]. In the present study, it is possible that the observed gender difference could be attributed to smaller sample size and fewer endpoints for females, thus increasing risk of Type 2 statistical error and should thus be interpreted with caution.

The strengths of the present study are that the study is registry based with complete national inclusion, have prospective registration of birth weight related variables, investigates a clinically useful condition (IgAN) and has a clinically relevant and reliable end point (ESRD). A weakness was that data on death was not available for the cohort and patients could not be censored for death. Another weakness is that ESRD is a rare outcome and usually takes considerable time to develop, number of patients with endpoints were therefore low in some subgroups. As an inclusion criterion of the study was being born after 1967, we were only able to include a relatively young cohort of IgAN patients (mean age at biopsy was 24 years). Previous studies have shown that for IgAN patients younger than 60 years and elevated serum creatinine level > 2.0 mg/dl, young age correlated with poor prognosis[72] [73].

Another limitation is that we did not have information regarding the treatment the patients received, most patients were however treated by nephrologists and received standard international care with angiotensin inhibition for those with either high blood pressure or proteinuria since the 1990’s, steroid treatment have been increasingly used since the early 2000’s for those with proteinuria above 1 gr/24h and preserved kidney function after a trial period with angiotensin inhibition.

In conclusion, the present study has shown that birth-weight related variables were associated with risk for developing ESRD, the association was stronger in male patients who were the predominant gender of this cohort (mirroring global trends). Both LBW and SGA were important risk markers, but the combination of these were especially important. Our results support the Brenner hypothesis. Future studies should investigate further the importance of SGA vs LBW and whether these markers are associated with altered renal morphology and risk of progression to ESRD also in other kidney diseases.

## Supporting Information

S1 Table Cohort characteristics at the time of IgAN diagnosis stratified by development of ESRD. (DOCX)

S2 Table Clinical and histopathological characteristics at the time of biopsy and risk of ESRD. (DOCX)
