# Supplementary material for: Low Birth Weight and Risk of Progression to End Stage Renal Disease in IgA Nephropathy—A Retrospective Registry-Based Cohort Study
Source: PLoS One. 2016 Apr 19;11(4):e0153819. doi: 10.1371/journal.pone.0153819 (PMC4836690; doi:10.1371/journal.pone.0153819)
Supplement: S1 Table — (DOCX) [file pone.0153819.s001.docx]

# **Supporting information**

S1Table. Cohort characteristics at the time of IgAN diagnosis stratified by development of ESRD.

|  | **ESRD** | | |
| --- | --- | --- | --- |
| **Clinicopathological characteristic** | **No** | **Yes** | **p-value** |
| N | 397 | 74 |  |
| N (%) male | 274(69.0%) | 58 (78.4%) | 0.1 |
| Age (years) | 23.9 ± 7.9 | 23.3 ± 6.3 | 0.5 |
| Systolic BP (mmHg) | 126.5 ± 19.0 | 142.7 ± 25.9 | < 0.001 |
| Diastolic BP (mmHg) | 76.6 ± 12.8 | 88.6 ± 16.9 | < 0.001 |
| eGFR (ml/min/1.73m^2^) | 105.9 ± 48.5 | 64.2± 35.5 | < 0.001 |
| Urinary protein (g/d) | 1.7 ± 2.1 | 3.7 ± 3.3 | < 0.001 |
| Proportion sclerosed glomeruli (%) | 0.08±0.16 | 0.17±0.22 | < 0.001 |
| Proportion with glomerular crescents (%) | 1.3 ± 5.5 | 2.3 ± 7.2 | 0.2 |
| Grade of interstitial fibrosis | 0.66 ± 0.66 | 1.4 ± 0.82 | < 0.001 |
| Grade of tubular atrophy | 0.53 ± 0.72 | 1.45 ± 1.01 | < 0.001 |
| N (%) birth weight <10^th^ percentile | 36 (9.1%) | 12 (16.2%) | 0.06 |
| N (%) gestational age <37 w | 21 (5.5%) | 6 (22.2%) | 0.3 |
| N (%) birth weight <10^th^ percentile for gestational age | 34 (8.9%) | 11 (16.2%) | 0.07 |
