# Supplementary material for: Low Birth Weight and Risk of Progression to End Stage Renal Disease in IgA Nephropathy—A Retrospective Registry-Based Cohort Study
Source: PLoS One. 2016 Apr 19;11(4):e0153819. doi: 10.1371/journal.pone.0153819 (PMC4836690; doi:10.1371/journal.pone.0153819)
Supplement: S2 Table — (DOCX) [file pone.0153819.s002.docx]

**Supporting information**

S2 Table. Clinical and histopathological characteristics at the time of biopsy and risk of ESRD.

| Variable name | Category definition | N total | N ESRD | HR (95% CI) | p-value | HR (95% CI)^a^ | p-value |
| --- | --- | --- | --- | --- | --- | --- | --- |
| Age (years) | <23.8 | 245 | 43 | 1.0(ref) |  | 1.0(ref) |  |
|  | ≥23.8 | 226 | 31 | 1.1(0.67-1.7) | 0.8 | 0.68(0.41-1.1) | 0.1 |
|  |  |  |  |  |  |  |  |
| Systolic BP (mmHg) | <140 | 316 | 34 | 1.0(ref) |  | 1.0(ref) |  |
|  | ≥140 | 123 | 34 | 3.3(2.0-5.3) | <0.001 | 2.3(1.4-3.8) | 0.001 |
|  |  |  |  |  |  |  |  |
| Diastolic BP (mmHg) | <90 | 335 | 35 | 1.0(ref) |  | 1.0(ref) |  |
|  | ≥90 | 104 | 33 | 4.2(2.6-6.8) | <0.001 | 3.2(2.0-5.2) | 0.001 |
|  |  |  |  |  |  |  |  |
| eGFR (ml/min/1.73m^2^) | >60 | 370 | 34 | 1.0 (ref) |  | 1.0 (ref) |  |
|  | 30-60 | 62 | 27 | 8.2 (4.8-13.8) | <0.001 | 8.2 (4.8-13.8) | <0.001 |
|  | <30 | 18 | 9 | 24.9 (11.3- 55) | <0.001 | 24.9 (11.3- 55) | <0.001 |
|  |  |  |  |  |  |  |  |
| Proteinuria (mg/24 h) | < 1 | 175 | 12 | 1.0(ref) |  | 1.0(ref) |  |
|  | 1-3 | 125 | 25 | 4.2(2.1-8.3) | <0.001 | 3.2(1.5-6.4) | 0.002 |
|  | ≥3 | 105 | 30 | 7.3(3.7-14.4) | <0.001 | 4.8(2.4-9.8) | <0.001 |
|  |  |  |  |  |  |  |  |
| Proportion of sclerosed glomeruli (%) | 0  1-19  ≥20 | 295  87  89 | 37  9  28 | 1.0(ref)  1.5(0.71-3.2)  6.1(3.5-10.5) | 0.3  <0.001 | 1.0(ref)  1.2(0.54-2.7)  3.0(1.6-5.5) | 0.7  0.001 |
|  |  |  |  |  |  |  |  |
| Glomerular crescents | No | 423 | 63 | 1.0(ref) |  | 1.0(ref) |  |
|  | Yes | 48 | 11 | 2.2 (1.1-4.1) | 0.02 | 1.5(0.76-2.9) | 0.1 |
|  |  |  |  |  |  |  |  |
| Grade of interstitial fibrosis ^b^ | none  focal mild  focal extensive | 186  212  66  7 | 10  31  27  6 | 1.0(ref)  3.3(1.6-6.8)  18.4(8.6-39.2)  191(61-599) | 0.001  <0.001  <0.001 | 1.0(ref)  3.8(1.7-8.3)  11.1(4.6-26.8)  74.2(19.5-282) | 0.001  <0.001  <0.001 |
| Grade of tubular atrophy ^c^ | none  mild  moderate  extensive | 250  142  61  18 | 16  21  25  12 | 1.0(ref)  3.5(1.8-6.8)  15.7(8.1-30.6)  43.5(19.6-96.2) | <0.001  <0.001  <0.001 | 1.0(ref)  3.3(1.7-6.6)  9.2(4.4-19.3)  17.5(7.2-42.3) | 0.001  <0.001  <0.001 |

^a^ Adjusted for estimated Glomerular Filtration in 3 categories (i.e. > 60, 30-59 and < 30 ml/min respectively)

^b^ Grade of interstitial fibrosis was categorized as mild, moderate or severe if the fibrosis involved <25%, 25-50% or more than 50% of the cortical area respectively.

^c^ Grade tubular atrophy was categorized as mild, moderate or severe if the tubular atrophy involved <25%, 25-50% or more than 50% of the cortical area respectively.
